# Supplementary material for: Measuring topology from dynamics by obtaining the Chern number from a linking number
Source: Nat Commun. 2019 Apr 15;10:1728. doi: 10.1038/s41467-019-09668-y (PMC6465319; doi:10.1038/s41467-019-09668-y)
Supplement: Supplementary file 1 — Supplementary Information [file 41467_2019_9668_MOESM1_ESM.pdf]

Measuring topology from dynamics by obtaining the Chern number from a linking number

M. Tarnowski *et al.*

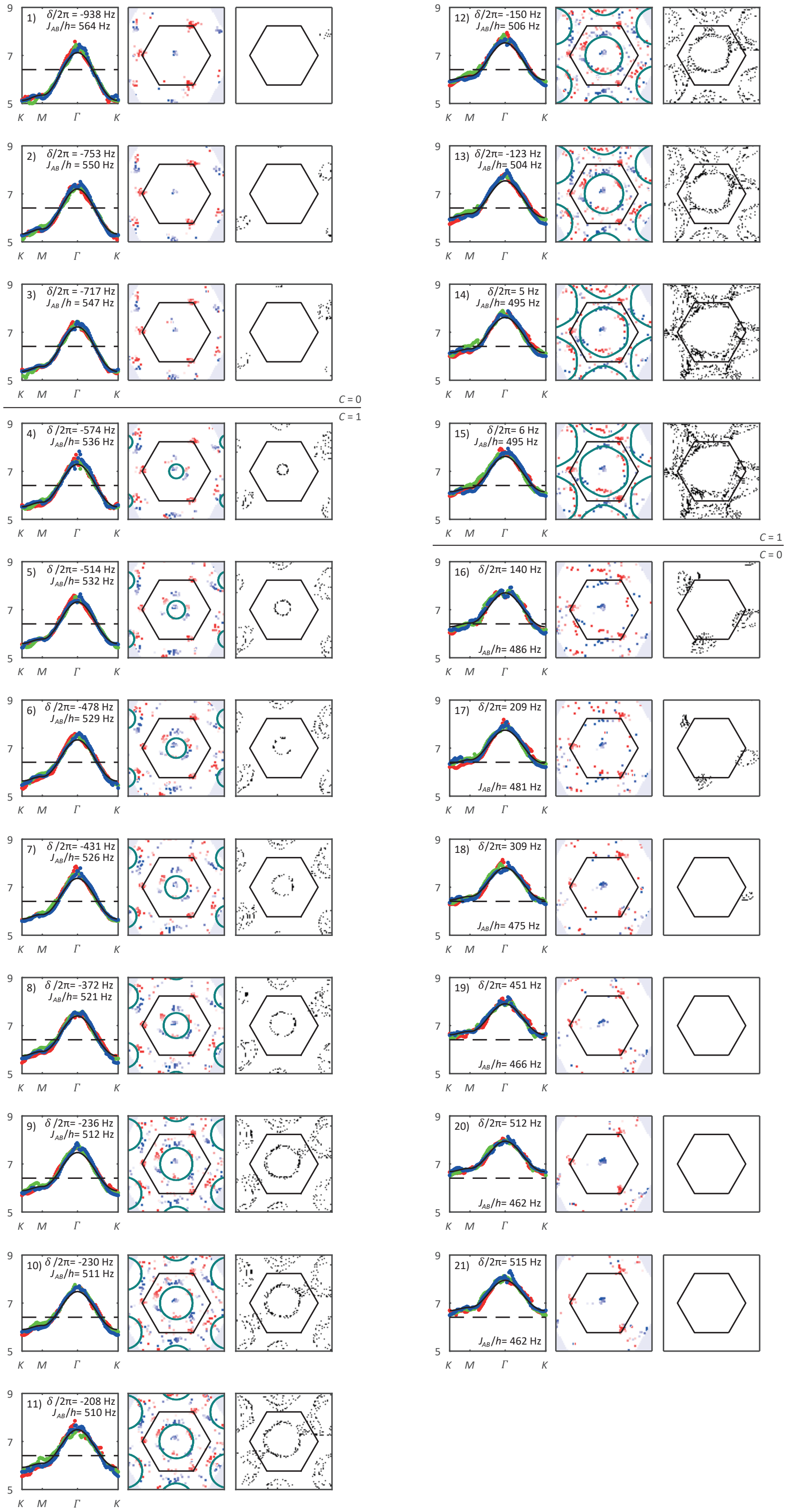

Supplementary Figure 1: Experimental data for different detunings and comparison to calculations. See text for details.

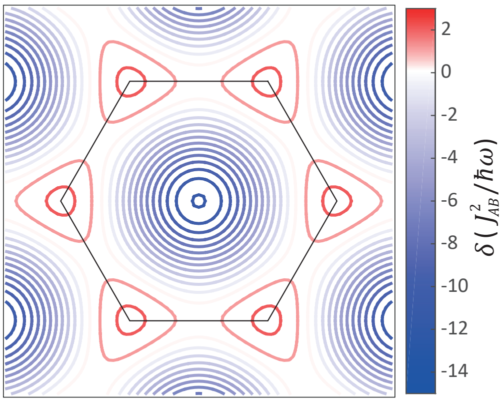

Supplementary Figure 2: Dynamical vortex contours. Countors of the dynamical vortices for different shaking detunings according to the simple model described in the text.

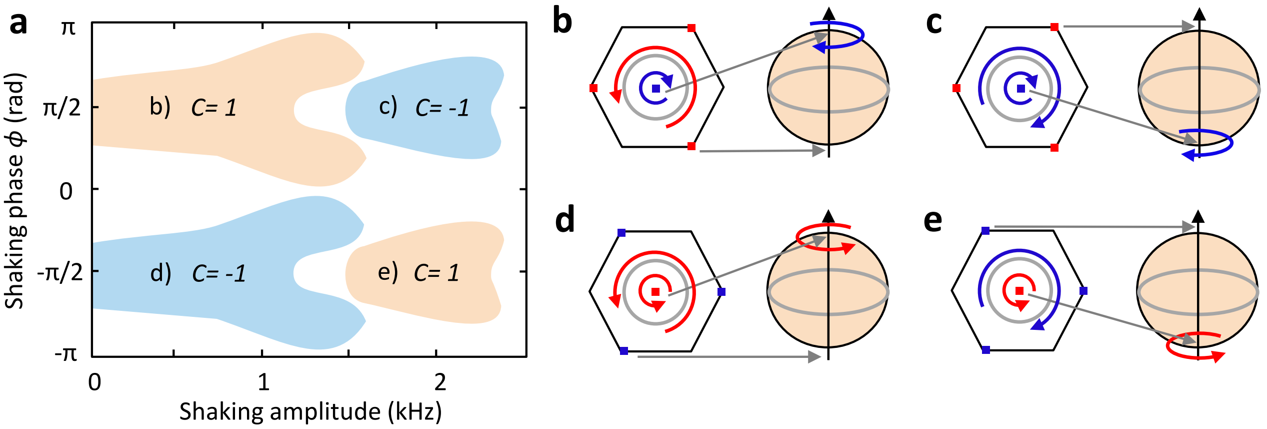

Supplementary Figure 3: Sign of the Chern number. **a** In our Floquet system, the sign of the Chern number can change either by inverting the shaking direction or by increasing the shaking amplitude. Phase diagram calculated for a detuning of $\delta/2\pi = -40\,\text{Hz}$. **b-e** Sense of wrapping the Bloch sphere in the four different cases.

Supplementary Note 1: Experimental data for different detunings

The experimental vortex data for different detunings leading to the phase diagram of Fig. 8 in the main text is presented in Supplementary Fig. 1. The different rows correspond to different detunings ranging from -938 Hz to 515 Hz. The detuning is varied by changing the lattice depth at fixed driving frequency of 6.410 kHz and driving amplitude of 1 kHz. The first column shows the band gap between the two lowest bare bands as obtained from the oscillation frequency of the tomography along three equivalent high symmetry paths (red, blue and green. The data is averaged over the six first time steps after the quench into the Floquet system). The collapse of the curves indicates the good balance of the three lattice beam intensities. The lattice parameters are obtained from a fit of the exact band structure to the band gap (black curve, regions around the static vortices are excluded from the fit). The extracted detuning and next-neighbor tunneling are stated in the respective subfigures. The dashed horizontal line indicates the shaking frequency and indicates the near-resonant nature of the driving.

The second column shows experimental data with the time-integrated static and dynamic vortices after the quench into the respective Floquet system. The hue indicates the time after the quench, at which the vortex appeared (lighter color means later time). The static vortices at the Γ and K points are present in all images. For non-trivial Chern number and in a regime of larger detunings, closed contours of dynamical vortices appear. The contour calculated from the effective Hamiltonian (green line) gives a reasonable approximation for the contour shape in the non-trivial regime.

The third column shows the expected dynamical vortices from a full numerical calculation including the initial state and the micromotion. Each black dot indicated a zero scalar product between the initial state and the time-evolved state.

Supplementary Note 2: Dynamical vortex contours from the effective Hamiltonian

The effective Hamiltonian allows deriving a simple estimate for the dynamical vortex contours (green lines in Supplementary Fig. 1). We neglect here the dispersion of the initial bands and the micromotion. In this approximation only non-trivial contours can be described, because the trivial contours arise from the finite dispersion of the initial bands. The vortex contour corresponds to the momenta, where the final Hamiltonian lies on the equator, i.e. where the z-component vanishes $h_z^{\mathrm{f}}(\mathbf{k}) = 0$. Using $h_z^{\mathrm{f}}(\mathbf{k}) = \Delta^{\mathrm{eff}}/2 + \sum_{j=1}^{3}(J_{AA}^{\mathrm{eff}} - J_{BB}^{\mathrm{eff}})\cos(\mathbf{k}\cdot\mathbf{b}_j)$ (compare the Hamiltonian derived in Methods) and the effective tunneling elements in the low driving limit, this corresponds to

$$S(\mathbf{k}) = \sum_{j=1}^{3}\cos(\mathbf{k}\cdot\mathbf{b}_j) = -\frac{\Delta^{\mathrm{eff}}}{2(J_{AA}^{\mathrm{eff}} - J_{BB}^{\mathrm{eff}})} = -\frac{\hbar\delta + 3J_{AB}^2/\hbar\omega}{4J_{AB}^2/\hbar\omega} = -\frac{\tilde{\delta}+3}{4} \quad (1)$$

with $\tilde{\delta} = \hbar\delta/(J_{AB}^2/\hbar\omega)$. The sum of the three cosines $S(\mathbf{k})$ can obtain values between $+3$ and $-3/2$. This means that contours only exist for detunings between $\tilde{\delta} = -15$ and $\tilde{\delta} = - + 3$, which defines the non-trivial region.

Supplementary Fig. 2 shows the value of the sum of the three cosines $S(\mathbf{k})$, which correspond to the vortex contours for different detunings $\tilde{\delta}$. The contour closes around the Γ point for $\tilde{\delta} = -15$ (where $S(\mathbf{k} = \Gamma) = 3$) and around the K and K' points for $\tilde{\delta} = +3$ (where $S(\mathbf{k} = K) = -3/2$). In Supplementary Fig. 1 the contours are plotted together with the data for the respective detunings (green lines). While we don't expect quantitative agreement on this level of approximation, the predictions qualitatively explain the behavior of the data in the non-trivial regime. Furthermore, this discussion gives an intuitive picture for the detunings, where the topological phase transitions occur.

Supplementary Note 3: Sign of the Chern number in our system

The sign of Chern number is given by the sense in which the Bloch sphere is covered. This is fixed by (i) which one of the two Dirac points is at the north pole of the Bloch sphere and (ii) in which sense the states wrap around the Bloch sphere azimuthally. These questions can be simply related to the chiralities of the observed static and dynamical vortices: the chirality of the dynamical vortex contour χ_{d} determines, which Dirac point is at the north pole, while the chirality of the enclosed static vortex χ_{s} determines the azimuthal winding of the states. For our choices of sign conventions, the sign of the Chern number of the lowest band is given by $\mathrm{sgn}[C] = -\chi_{\mathrm{d}}\chi_{\mathrm{s}}$.

The four different possible combinations of $\chi_{\mathrm{d}} = \pm 1$ and $\chi_{\mathrm{s}} = \pm 1$ can be realized in our system by changing the shaking parameters: (i) the occupation of the poles by the Dirac points inverts for large shaking amplitudes and (ii) the sense of wrapping around azimuthally inverts with the direction of shaking. Data for the two shaking directions at small shaking amplitude is presented in Fig. 9 of the main text. Supplementary Fig. 3 illustrates the four different possibilities in the phase diagram spanned by shaking phase and shaking amplitude.

From the analysis of χ_{d} and χ_{s}, one can therefore also distinguish whether the Chern number changes sign due to a different shaking direction or due to a large driving amplitude, where the sign change of the Chern number originates from the sign change of the Bessel function renormalization of the tunnel elements. Such phases have been realized in cold atoms [1] and helical photonic waveguides [2].

Supplementary References

[1] N. Fläschner, D. Vogel, M. Tarnowski, B. S. Rem, D. S. Lühmann, M. Heyl, J. C. Budich, L. Mathey, K. Sengstock, and C. Weitenberg, *Observation of dynamical vortices after quenches in a system with topology*, Nature Physics **14**, 265 (2018).

[2] J. Guglielmon, S. Huang, K. P. Chen, and M. C. Rechtsman, *Photonic realization of a transition to a strongly driven Floquet topological phase*, Phys. Rev. A **97**, 031801(R) (2018).
